# Potential therapeutic effects of traditional Chinese medicine in acute mountain sickness: pathogenesis, mechanisms and future directions

**DOI:** 10.3389/fphar.2024.1393209

**Published:** 2024-06-04

**Authors:** Zhenhui Wu, Yihao Wang, Rong Gao, Junru Chen, Yingfan Chen, Maoxing Li, Yue Gao

**Affiliations:** ^1^ School of Pharmacy, Jiangxi University of Chinese Medicine, Nanchang, China; ^2^ Department of Hematology, Affiliated Hospital of Jiangxi University of Chinese Medicine, Nanchang, China; ^3^ Beijing Institute of Radiation Medicine, Beijing, China; ^4^ Department of Traditional Chinese Medicine, The Sixth Medical Center of Chinese People’s Liberation Army General Hospital, Beijing, China

**Keywords:** traditional Chinese medicine, acute mountain sickness, hypobaric hypoxia, pathogenesis, mechanism

## Abstract

**Background and objectives:**

Acute mountain sickness (AMS) is a pathology with different symptoms in which the organism is not adapted to the environment that occurs under the special environment of high altitude. Its main mechanism is the organism’s tissue damage caused by acute hypobaric hypoxia. Traditional Chinese medicine (TCM) theory focuses on the holistic concept. TCM has made remarkable achievements in the treatment of many mountain sicknesses. This review outlines the pathogenesis of AMS in modern and traditional medicine, the progress of animal models of AMS, and summarizes the therapeutic effects of TCM on AMS.

**Methods:**

Using the keywords “traditional Chinese medicine,” “herbal medicine,” “acute mountain sickness,” “high-altitude pulmonary edema,” “high-altitude cerebral edema,” “acute hypobaric hypoxia,” and “high-altitude,” all relevant TCM literature published up to November 2023 were collected from Scopus, Web of Science, PubMed, and China National Knowledge Infrastructure databases, and the key information was analyzed.

**Results:**

We systematically summarised the effects of acute hypobaric hypoxia on the tissues of the organism, the study of the methodology for the establishment of an animal model of AMS, and retrieved 18 proprietary Chinese medicines for the clinical treatment of AMS. The therapeutic principle of medicines is mainly invigorating qi, activating blood and removing stasis. The components of botanical drugs mainly include salidroside, ginsenoside Rg1, and tetrahydrocurcumin. The mechanism of action of TCM in the treatment of AMS is mainly through the regulation of HIF-1α/NF-κB signaling pathway, inhibition of inflammatory response and oxidative stress, and enhancement of energy metabolism.

**Conclusion:**

The main pathogenesis of AMS is unclear. Still, TCM formulas and components have been used to treat AMS through multifaceted interventions, such as compound danshen drip pills, Huangqi Baihe granules, salidroside, and ginsenoside Rg1. These components generally exert anti-AMS pharmacological effects by inhibiting the expression of VEGF, concentration of MDA and pro-inflammatory factors, down-regulating NF-κB/NLRP3 pathway, and promoting SOD and Na + -K + -ATPase activities, which attenuates acute hypobaric hypoxia-induced tissue injury. This review comprehensively analyses the application of TCM in AMS and makes suggestions for more in-depth studies in the future, aiming to provide some ideas and insights for subsequent studies.

## Graphical Abstract

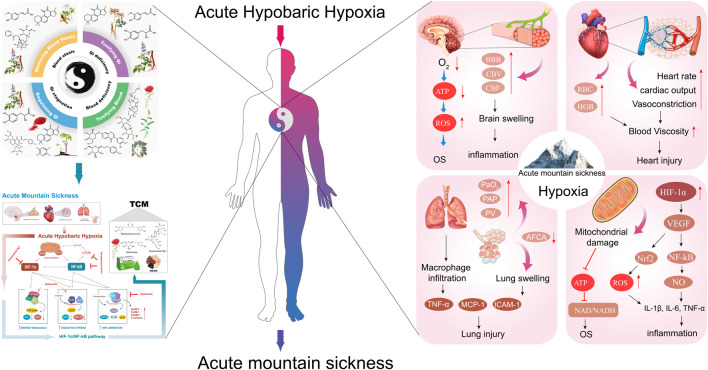

## 1 Introduction

China is the country with the largest plateau area in the world, and its area with an altitude greater than 2500 m accounts for more than a quarter of its land area. Compared with the plains, the high altitude condition has the characteristics of low air pressure, low oxygen partial pressure, coldness, strong ultraviolet rays, and dry climate. Among them, hypoxia has non-specific effects on the body’s functions and metabolism and is the most important factor affecting the human body in the high altitude conditions (Richalet et al., 2023). Acute mountain sickness (AMS) refers to the condition that occurs due to low oxygen when people rush from plain areas or low-altitude areas to high-altitude areas. It is mainly headache, and may be accompanied by dizziness, loss of appetite, and nausea. A clinical syndrome with symptoms such as vomiting, palpitation, insomnia, fatigue, and fatigue (Burtscher et al., 2023). From the perspective of medical altitude (high altitude), 3,000 m above sea level was once used as the plateau limit. However, at the sixth International Conference on High Altitude Medicine held in Xining, Qinghai in 2004, 2,500 m above sea level was redefined as the medical altitude limit. Once one rushes from the mainland to an area above 2,500 m above sea level, AMS may occur (Wu, 2005; Luks et al., 2010). Although AMS is not fatal in most cases, in severe cases, it can progress to high altitude pulmonary edema (HAPE) and high altitude cerebral edema (HACE), both of which are also called acute severe mountain sickness. Generally not self-healing and can be life-threatening (West, 2012; Luks et al., 2017).

Currently, a large number of people travel from plain areas to high altitude areas for tourism, work, sports competitions and performing tasks. The incidence of AMS is closely related to altitude. According to statistics, the incidence rate of AMS in people who have not acclimated to hypobaric hypoxia is about 10%–25% after they quickly arrive at a high-altitude of 2500 m from the plains, and the symptoms are mild. The incidence rate of AMS after quickly reaching high altitude areas of 4500–5500 m is about 50%–85% and may affect mobility. In severe cases, it can cause severe altitude sickness such as HAPE and HACE (Norris et al., 2012; Garrido et al., 2021). Therefore, it is necessary to conduct in-depth research on the pathogenesis and drug treatment for AMS. The overall occurrence of AMS among participants was 73.5% (23.2% mild, 50.3% moderate–severe) (Cobb et al., 2021). Eleven of 21 volunteers had AMS and lower resting Blood oxygen saturation (SaO2) levels while ascending to high altitude (Fayazi et al., 2023).

Under acute hypobaric hypoxia, the body adapts to the external environment by changing a series of gene expressions, and hypoxia inducible factor-1 (HIF-1) is the most important transcription factor that regulates cellular adaptation to hypoxia, which plays a key role in cellular perception and acclimatation to the change of oxygen concentration (Li et al., 2020a). HIF-1 is a heterodimer consisting of two subunits: HIF-1α and HIF-1β. Under normoxia, HIF-1 was hydroxylated by oxygen and proline hydroxylase (PHD), and the hydroxylated modified HIF-1α was rapidly degraded after binding to oncoproteins, resulting in the inability of HIF-1α to be induced to be expressed (Bw et al., 2013). In hypobaric hypoxia, respiratory metabolism is enhanced, increasing reactive oxygen species (ROS) radicals in the electron transport chain complex III, which inactivates PHD, and the inactivated PHD can affect the stability of HIF-1α protein through the phosphorylation pathway (Bw et al., 2013). Additionally, under acute hypobaric hypoxia, degradation of HIF-1α is reduced, allowing it to stabilize and accumulate in the cell, where it then binds to HIF-1β and translocates to the nucleus, activating multiple downstream genes (Dzhalilova and Makarova, 2020). These genes are involved in promoting erythropoiesis (increased production of the erythropoietin EPO), increasing angiogenesis (upregulation of the vascular endothelial growth factor VEGF), and reprogramming cellular energy metabolism (increased expression of glycolytic enzymes) (Lappin and Lee, 2019). The mechanisms of HIF-1a involved in acute hypobaric hypoxia include the ROS pathway, the Toll-like receptor 4 (TLR4) signaling pathway, and the autophagy pathway (El Alam et al., 2022). Targeted inhibition on different pathways may be effective in attenuating acute hypobaric hypoxia-induced multiple tissue injuries.

With its multi-compounds and multi-target properties, traditional Chinese medicine (TCM) has been widely used as an alternative medical treatment for various diseases (Gan et al., 2023). Studies have shown that TCM has good clinical efficacy in treating AMS, and can significantly increase the levels of hemoglobin, hematocrit and antioxidant factors, and improve the ability to adapt to high altitude exposure (Li et al., 2020b). Therefore, this paper reviews the effects of acute hypobaric hypoxia on the organism, the pathogenesis of AMS, animal models of AMS, and the efficacy and mechanism of action of Chinese medicine in the treatment of AMS.

## 2 Methods

Literature searches were conducted through Scopus, Web of Science, PubMed, and China National Knowledge Infrastructure databases using the terms “traditional Chinese medicine,” “herbal medicine,” “acute mountain sickness,” “high-altitude pulmonary edema,” “high-altitude cerebral edema,” “acute hypobaric hypoxia,” and “high-altitude,” as well as various combinations of these keywords. We reviewed the retrieved literature for the pathogenesis of AMS and the efficacy and mechanism of action of TCM on AMS. Relevant studies on the mechanisms of AMS since the establishment of the repository up to November 2023 were selected to ensure completeness of the review and to provide support.

### 2.1 Inclusion criteria

1) Clinical and animal studies of TCM in relation to AMS, HAPE, HACE; 2) Studies on acute high altitude hypobaric hypoxia associated with TCM; 3) Clinical study of TCM for AMS.

### 2.2 Exclusion criteria

1) Studies related to simple hypoxia; 2) AMS-related questionnaire survey and Meta-analysis studies; 3) Clinical studies on non-Chinese medicine related drugs for AMS treatment.

## 3 The pathogenesis of AMS

### 3.1 Mechanisms of hemodynamic and hemorheological stress to acute hypobaric hypoxia

**FIGURE 1:**
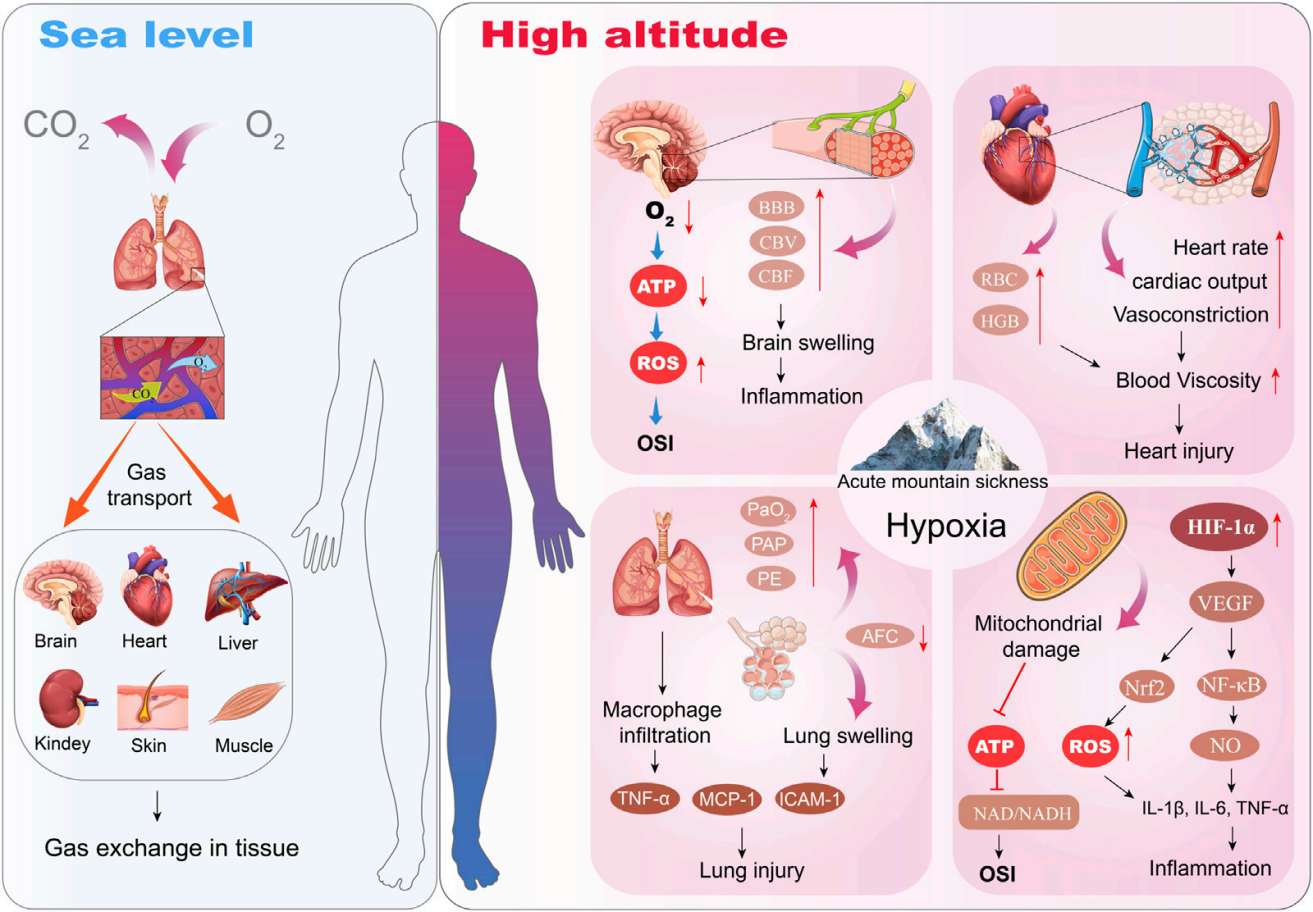
Effects of Acute Hypobaric Hypoxia in the development of AMS.

Under acute hypobaric hypoxia, the body will naturally increase cardiac output to ensure the supply of oxygen to vital organs, resulting in a faster heartbeat and higher blood pressure. At the same time, to increase the efficiency of oxygen transport, the red blood cells in the blood will increase, making the blood viscosity increase, leading to a decrease in the speed of blood flow, which in turn affects the delivery of oxygen (Richalet et al., 2023). The increased blood viscosity will also lead to a decrease in the deformation ability of red blood cells and impeded blood circulation. Particularly in the microvasculature, this may lead to microcirculatory disturbances that affect the supply of oxygen and nutrients to the tissues. In addition, cerebral vasodilatation occurs in hypobaric hypoxia environments to increase cerebral blood flow to compensate for the lack of oxygen, but this may lead to damage to the blood-brain barrier, resulting in elevated intracranial pressure, as shown in Figure 1. Elevated intracranial pressure, if not properly controlled, can further progress to HACE (Wilson et al., 2009; Turner et al., 2021). This increase in intracranial pressure is a key cause of major AMS symptoms such as headache, nausea, fatigue, and insomnia. In addition to this, due to the hypoxic environment, the pulmonary blood vessels also constrict, which can lead to an increase in pulmonary blood pressure, and in the long term may result in the formation of HAPE (El Alam et al., 2022).

### 3.2 Changes in inflammatory cytokines

Hypobaric hypoxia is known to regulate inflammation (Bhattacharya et al., 2021). Inflammation is considered an important risk factor for the occurrence of AMS, and it plays a crucial role in the physiological response to hypoxia (El Alam et al., 2022; Pham et al., 2022). Hypobaric hypoxia exposure can lead to changes in the inflammatory profile (Pham et al., 2022). Studies have shown that the concentrations of pro-inflammatory cytokines and inflammatory markers such as C-reactive protein, interleukin-1β (IL-1β), IL-6, IL-17F, and CCL8 in acutely exposed individuals at high altitudes are significantly higher than at sea level, while the concentrations of the anti-inflammatory cytokine IL-10 are significantly lower than at sea level (Song et al., 2016; Liu et al., 2017). Meanwhile, in the HACE model induced by low-dose lipopolysaccharide (LPS, 0.5 mg/kg) combined with acute hypobaric hypoxia exposure (6000 m, exposure for 24 h), the level of TNF-α, IL-1β, and IL-6 in plasma and hippocampal tissue increased significantly, and the brain water content increased. These results demonstrate that hypobaric hypoxia augments LPS-induced inflammation and induces the occurrence and development of HACE in mice (Zhou et al., 2017).

In addition, Pham et al. showed that several inflammation-related genes were significantly upregulated in participants' peripheral blood after 1 day of hypobaric hypoxia exposure (3800 m), including HMGB1 (high mobility group box 1), calprotectin (S100A8), TLR4 and LY96 (Immunogenic cell death-related hub genes) (Pham et al., 2022). HMGB1 is a damage-associated molecular pattern (DAMP) molecule that amplifies immune responses during tissue damage (Pham et al., 2022). Additionally, hypobaric hypoxia can stimulate immune cells, such as macrophages and lymphocytes, to release a variety of inflammatory mediators, including tumor necrosis factor-alpha (TNF-α), IL-1, IL-6 and nitric oxide (NO), as shown in Figure 1. These inflammatory factors not only increase vascular permeability, leading to edema; they may also trigger systemic or local inflammatory responses (El Alam et al., 2022). The prolonged inflammatory response may also lead to tissue damage, further exacerbating the severity of acute mountain sickness. It has been shown that acute hypobaric hypoxia responders have higher levels of IL-1 receptor agonist (IL-1RA), heat-shock protein 70 (HSP-70), and adrenomedullin (Julian et al., 2011). In addition, macrophage inflammatory protein 1β (IL-1β) was higher in patients with acute hypobaric hypoxia response compared to the non-plateau-responsive population (Wang et al., 2018), suggesting that prevention of acute high altitude condition response may be aided by anti-inflammation. Bhattacharya et al. explored potential targets for regulating inflammation by genotyping the whole blood of Tibetan population in hypobaric hypoxia exposure areas, and proposed that enhancing PHD2 activity may be an important method to reduce inflammation caused by hypoxia (Bhattacharya et al., 2021). Therefore, inhibiting the inflammation caused by hypoxia may be used as a therapeutic strategy to reduce the occurrence of AMS disease.

### 3.3 Oxidative stress injury

Oxidative stress can cause an inflammatory response, and in turn, the inflammatory response itself can also trigger oxidative stress, and both promote and influence each other (Liao et al., 2023). Hypobaric hypoxia exposure-induced oxidative stress is a key pathological mechanism for AMS (Li et al., 2024). Oxidative stress injury is ubiquitous in the pathogenesis of AMS, HACE, and HAPE, and has become a hot topic of research in hypobaric hypoxic in recent years (Pena et al., 2022). Studies have shown that when exposed to acute hypobaric hypoxic (above 4500 m), the pO2 gradient decreases dramatically, and oxygen can activate the oxidative stress response by regulating the STAT3-RXR-Nrf2 signaling pathway, promoting reactive oxygen species (ROS) generation (Paul et al., 2018). Interestingly, high altitude hypoxia increases endoplasmic reticulum stress and oxidative stress (Hu et al., 2021). Whereas hypoxia-induced oxidative stress mediates endoplasmic reticulum dysfunction, which leads to the accumulation of unfolded proteins (Pena et al., 2022); this dysregulation contributes to the acceleration of multiple processes associated with hypoxic inflammatory pathways. Additionally, Himadri et al. found that rats exposed to a simulated altitude of 25,000 ft for 24 h developed HACE, with increased levels of ROS and MDA, decreased levels of SOD and GSH-Px, increased oxidative stress, and upregulation of NF-κB in brain tissues (Himadri et al., 2010). Moreover, oxidative stress and immune responses are rapidly initiated in rats under hypobaric hypoxic stress; however, with prolonged hypoxic stress, excessive oxidative stress further stimulates the immune system in the body and releases large amounts of inflammatory factors that accumulate in the body (Pu et al., 2022). This, in turn, may lead to an inflammatory storm and further damage to lung tissue resulting in AMS (Pu et al., 2022). Therefore, eliminating oxidative stress with an anti-oxidative and anti-inflammatory agent could be an important strategy to use as prophylaxis and in the treatment of AMS (Pena et al., 2020; Pena et al., 2022).

### 3.4 Autophagy

Hypoxia induces autophagy, which plays an important role in the protection of the body against tissue damage. Hu Y et al. found that prolonged high altitude hypoxia can lead to enhanced basal autophagy (Hu et al., 2021). Autophagy flux was increased in high altitude hypoxic environment, as evidenced by an increase in the ratio of microtubule-associated protein 1 light chain type 3 II/I (LC3-II/I) and an increase in the expression of LC3-II (Masschelein et al., 2014). In addition, Zhao Z et al. also found that prolonged exposure to high altitude hypoxia resulted in increased lung injury, formation of autophagosomes with a double-membrane structure, and increased levels of Beclin-1 and LC3-II in alveolar tissues compared with controls (Zhong et al., 2022). However, autophagy is a double activity. Acute hypobaric hypoxia may also inhibit levels of autophagy. Interestingly, using a hypoxic chamber (4000 m, 56.0 kPa, 6000 m, 47.0 kPa, and 8000 m, 36.0 kPa), Dai S et al. demonstrated that acute hypoxic exposure impaired autophagic activity in early and late stages of the mouse brain and partially contributed to hypoxia-induced oxidative stress, neuronal loss, and brain damage (Dai et al., 2024). This result confirms that enhanced autophagy activity attenuates neurological deficits after oxidative stress and exposure to hypobaric hypoxia (Dai et al., 2024). Therefore, whether to inhibit autophagy or to promote autophagy levels for the treatment of tissue damage induced by high altitude hypoxia requires an individualised comprehensive analysis.

### 3.5 Blood-brain barrier damage (increased cerebral vascular permeability)

The development of AMS is considered to be a multifactorial process, and blood-brain barrier (BBB) dysfunction caused by acute hypobaric hypoxia and the resulting vasogenic edema are considered to be one of the potential mechanisms. The BBB is a highly selective semipermeable membrane barrier that exists between the brain and blood and maintains homeostasis in the brain’s internal environment (Neuwelt et al., 2011). The function of the BBB depends on the integrity of the neurovascular unit (NVU), which consists of endothelial cells, perivascular astrocytes, neurons, and microglia (Hourfar et al., 2023). These tight junctions between cells are essential for maintaining the integrity of the BBB and these tight junctions are regulated by several types of proteins such as intracellular occluder band-1 (ZO-1), occludin, claudins, and junctional adhesion molecules (Luo et al., 2024). Disruption of the tight junction induced by hypoxia is an important cause of BBB impairment and cerebral edema (Chen et al., 2020). Hypoxia-induced disruption of endothelial tight junctions triggers BBB injury and induces vasogenic edema (Xue et al., 2022). Studies have shown that hypoxia (1% O2 for 24 h) upregulates CAV-1 transcription through activation of NRF1 in endothelial cells, which induces internalization of claudin-5 and autophagic degradation, and accordingly, these effects lead to BBB disruption and trigger HACE (Xue et al., 2022). Moreover, hypoxia enhances lipopolysaccharide (LPS)-induced inflammatory responses and triggers HACE in mice (Zhou et al., 2017). LPS-induced systemic inflammatory responses rapidly exacerbated cerebral edema after acute hypobaric hypoxia exposure by disrupting the integrity of the blood-brain barrier and activating microglia, and by increasing water permeability through the accumulation of aquaporin-4 (AQP4) (Zhou et al., 2017). Furthermore, NB-3 (contactin-6) is a neural recognition molecule that also plays a key role in the development of HACE. Studies have shown that NB-3 is expressed in neurons and endothelial cells, and that deletion of NB-3 decreases levels of tight junction proteins, which disrupts BBB integrity and exacerbates HACE and BBB leakage (Zhou et al., 2017).

### 3.6 Regulation of related signaling pathways

#### 3.6.1 Inflammatory signaling pathways

Hypobaric hypoxia also activates multiple inflammation-related signaling pathways. These include the nuclear factor-κB (NF-κB) pathway and the NOD-like receptor thermal protein domain associated protein 3 (NLRP3) pathway, which are commonly associated with stress and inflammatory responses (Jiang et al., 2022; Pu et al., 2022). NF-κB is an important intracellular transcription factor that is essential for regulating inflammatory responses. Under hypobaric hypoxia conditions, NF-κB can be activated and translocated to the nucleus, activating a variety of inflammatory response genes, such as cytokines, chemokines, and adhesion molecules (Pena et al., 2020). An increase in these factors can lead to an inflammatory response in cells and tissues, promoting leukocyte infiltration and producing edema. Zeng et al. found that acute hypobaric hypoxia (6000 m, exposure for 3 days) induced a significant increase of TLR4, NLRP3, p-NF-κB p65, caspase-1 protein and gene expressions in lung tissue samples and induced acute lung injury (Zeng et al., 2024). Additionally, Jia et al. found that NLRP3, gasdermin D (GSDMD), cleaved caspase-1, and cleaved IL-1β levels were upregulated in acute hypobaric hypoxia rats (6000 m, exposure for 3 days), suggesting that acute hypobaric hypoxia could activate the NLRP3/caspase-1 signaling pathway in heart tissue of acute hypobaric hypoxia-induced heart injury rats (Jia et al., 2023). Mechanistically, acute hypobaric hypoxia (6000 m, exposure for 3 days) aggravates oxidative stress and upregulates (pro)-inflammatory cytokines, activating NLRP3 inflammasome-mediated pyroptosis (Shen et al., 2023). Similaryly, hypobaric hypoxia (5000 m, exposure for 7 days) can induce retinal pyroptosis in rats by inducing activation of NLRP3 inflammasome and release of proinflammatory cytokines (Xin et al., 2022).

#### 3.6.2 Hypoxia-sensing and hydrogen sulfide pathways

Cellular sensing and adaptation to changes in external oxygen concentration are important active processes in living organisms. Oxygen homeostasis is essential for the maintenance of body function. The disruption of oxygen homeostasis can lead to abnormal biological functions such as gene expression, energy metabolism, vascular regeneration, and stem cell proliferation and differentiation (Ghoneum et al., 2020). Carotid body is a sensory organ that monitors the concentration of oxygen in arterial blood. Decreased oxygen concentration in arterial blood stimulates neural activity in the carotid body, triggering reflex stimulation of respiration and blood pressure, which are essential for the maintenance of homeostasis in a hypoxia environment (Peng et al., 2023). Hydrogen sulfide (H2S) signaling is associated with hypoxic activation of carotid bodies (Peng et al., 2023). It is suggested that H2S is involved in sensing and response to hypoxia in multi-tissues that possess the ability to sense hypoxia (Wu et al., 2015). Using a hypobaric hypoxia animal model, it was demonstrated that exposure of animals to hypobaric hypoxia (7620 m, exposure for 1, 3, and 7 days) resulted in a significant decrease in H2S levels in the brain (Mishra et al., 2020). After exposure to acute hypobaric hypoxia (5260 m, for 1 day), H2S levels decreased significantly, while hypobaric hypoxia promoted glycolysis and dysregulated the pentose phosphate pathway, as well as purine catabolism, glutathione homeostasis, arginine/nitric oxide and sulfur/H2S metabolisms (D’Alessandro et al., 2016). Under hypobaric hypoxia exposure conditions (7620 m, 8% O2, for 7 days), increasing H2S levels inhibited hypobaric hypoxia-induced apoptosis in hippocampal neurons and also significantly protected glial vascular homeostasis and key neurophysiological functions (such as cerebral blood flow, functional congestion, and spatial memory) (Kumar et al., 2016a).

Mechanistically, H2S regulates intracellular O2 homeostasis and inhibits hypoxia-induced HIF-1 activation in a VHL- and mitochondria-dependent manner (Kai et al., 2012). Reduction of endogenous H2S increased pulmonary respiratory membrane permeability in HAPE rats and leads to increased protein exudation (Wang et al., 2017). This suggests that the reduction of H2S *in vivo* exacerbates the damage to lung respiratory membranes caused by acute hypobaric hypoxia. Moreover, Yang et al. found that in the early stage of exposure to acute hypobaric hypoxia, the stress of plasma H2S production was increased and pulmonary arterial pressure was elevated, suggesting that exogenous supplementation of H2S could delay acute hypobaric hypoxia-induced pulmonary arterial pressure elevation (Yang et al., 2011). Therefore, H2S can be used as a promising molecule to provide a reference for the development of AMS drugs.

## 4 Animal model construction of AMS, HACE, and HAPE

AMS is mainly caused by acute hypobaric hypoxia stimulation (Irarrázaval et al., 2017). Therefore, the animal model of AMS can be established in high altitude condition simulation chamber or plateau field. The model can be established by feeding in the hypobaric hypoxic chamber or animal treadmill-assisted sports training (Huo et al., 2021). Blood oxygen saturation (SaO2), blood gas, inflammatory factors, tissue water content, and pathological sections are commonly used to evaluate the success of the animal model (Wang et al., 2018; Huo et al., 2021; Peng et al., 2022).

Interestingly, some of the animal experimental conditions for high-altitude hypobaric hypoxia injury are consistent with the AMS animal model. The difference is the final detection index and evaluation method. Zeng Y et al. established an animal model of lung injury from hypobaric hypoxia in a high altitude condition, which involved exposing SD rats to hypoxia at an altitude of 6000 m, with an ascent rate of 10 m/s, and continuous exposure to hypoxia for 3 days (Zeng et al., 2024). Additionally, Zhang P et al. chose cynomolgus monkeys to establish an acute hypobaric hypoxia brain injury model, and the specific steps: altitude simulation was started at 320 m, then suspended at 3000 m, 4500 m, and 6000 m for 50 min each, and finally maintained at 7500 m for an initial 48 h, with a rate of ascent of 3 m/s (Zhang et al., 2021). Detailed AMS animal model construction methods and animal type information are shown in Table 1.

**TABLE 1:** Animal model construction of AMS, HACE, and HAPE.

Indication	Altitude	Ascent rate	Descent rate	Exposure time	Animal	Evaluation of indicators	Ref
AMS	5000 m	10 m/s	20 m/s	24 h	Male SD rats	SaO2 ↓, PaO2 ↓, heart Hematoxylin-eosin (HE) staining, RBC ↑, HGB ↑	Yan et al. (2022)
AMS	7000 m	10 m/s	10 m/s	7 days	Male SD rats	Heart water content ↑, heart HE staining	Shi et al. (2019)
AMS	5000 m, 6000 m	2 m/s	2 m/s	12, 24, 48 h	Male Wistar rats	PO2 ↓, brain and lung HE staining	Wang et al. (2015)
AMS	6000 m	10 m/s	20 m/s	6–12 h	Male BALB/C mice	Brain and heart HE staining	Ma et al. (2013)
AMS	7000 m	—	—	24 h	Male C57BL/6J mice	Recognition memory (discrimination ratio ↓), Open field test (total distance ↓, central distance ↓)	Wang et al. (2022)
AMS	6000 m	—	—	24 h	Male SD rats	Heart tissue HE staining	Ma et al. (2022)
AMS	6000 m	—	—	24, 48, 72 h	Male SD rats	Lung tissue HE staining	Pu et al. (2022)
AMS	8229.6 m	5 m/s	—	12 h	Male SD rats	Cerebral vascular leakage ↑, brain HE staining	Kumar et al. (2016b)
AMS	5000 m, 8000 m, 10000 m	16.67 m/s	—	5000 m and 8000 m maintained for 5 min, and 10,000 m maintained for 1 h	BALB/C mice	Survival time ↓, lactic acid ↑, lactate dehydrogenase content ↑	Ma et al. (2011)
AMS	3600 m, 5000 m	—	—	goats (3600 m); rabbits (5000 m)	goats (48 h); rabbits (4 h)	Blood Gases (SaO2 ↓, PaO2 ↓, lactic acid ↑), Brain and Lung water content ↑	Zhang et al. (2021)
HACE	6000 m	50 m/s	—	3 days	Male C57BL/6J mice	Brain water content ↑, BBB permeability ↓, brain HE staining	(Huang et al., 2015)
HACE	4000 m, 8000 m	—	—	4000 m (2days treadmill adaptation), 8000 m (3days)	Male SD rats	BBB permeability ↓, brain HE staining	(Guo et al., 2013)
HACE	7000 m	—	—	3 days	Male SD rats	Brain water content ↑, vascular leakage ↑, GSH ↓, SOD ↓, and MDA ↑	Su et al. (2021)
HACE	6000 m	50 m/s	—	6 h (LPS stimulation)	Male C57BL/6J mice	BBB integrity ↓, inflammatory factor ↑	(Geng et al., 2022)
HAPE	4700	20 m/s	—	48 h (exercise)	Male SD rats	Arterial blood gas (PaCO2 ↓, PaO2 ↓), bronchoalveolar lavage ↑, lung wet-to-dry weight ratio ↑, HE staining	(Bai et al., 2010)
HAPE	7000 m	10 m/s	—	48 h (cold)	Male SD rats	Lung water content ↑, lung HE staining, lung wet-to-dry weight ratio ↑	Yang et al. (2011)
HAPE	6000 m	10 m/s	—	72 h	Male SD rats	Lung water content ↑, lung HE staining, lung wet-to-dry weight ratio ↑	Yang et al. (2011)

Note: AMS, acute mountain sickness; BBB, blood brain barrier; GSH-PX, glutathione peroxidase; HE, Hematoxylin-eosin; HGB, hemoglobins; MDA, malondialdehyde; PaO_2_, partial arterial oxygen pressure; PaCO_2_, CO_2_ partial pressure; RBC, red blood cell; SaO_2_, blood oxygen saturation; SOD, Superoxide dismutase.

## 5 Traditional Chinese medicine in the treatment of AMS

### 5.1 Understanding AMS in traditional Chinese medicine theory

The body inhales insufficient pure air from the external high altitude conditions, which can easily lead to shortness of qi or qi deficiency in the body. In traditional Chinese medicine (TCM) theory, “qi” is an important concept, which refers to the manifestation of life activity and vitality and is one of the basic substances that make up everything in the universe. When stepping into the high altitude condition from the sea level, the body is suddenly stimulated by the cold and lack of oxygen, the body’s zongqi is disordered, the spleen and stomach cannot be adequately moistened, nausea and vomiting easily. Insufficient qi in the high altitude condition results in insufficient zong qi, creating qi deficiency. Qi deficiency leads to blood stasis and insufficient blood supply to the brain, resulting in weakness and tiredness, and attacks of vertigo (Gao et al., 2023). TCM theory suggests that the etiology of this disease is a combination of qi deficiency, blood stasis, dampness stagnation, and phlegm and blood stasis obstruction (Feng et al., 2013). Most doctors believe that the cause of AMS is mainly divided into external and internal causes. The external causes are caused by the terrestrial qi and the celestial qi, which include lack of pure air, severe cold, and dry climate in the altitude. The internal causes are caused by the deficiency of qi in the human body (Zhang and Zhang, 1993; Li et al., 2012; Feng et al., 2013).

### 5.2 Understanding AMS in Tibetan medicine’s theory

Although the concept of altitude diseases is not explicitly mentioned in the ancient books of Tibetan medicine, Tibetan medicine itself comes from the actual life of altitude people, so its essence is altitude medicine. Tibetan medicine is a great medical system formed by the people in the high-altitude hypoxia region in the struggle against various diseases and harsh natural environments (Liu et al., 2018a).

According to Tibetan medical theory and “Four Tantras”《四部医典》, there are three major factors in the human body ("Dragon”, “Chiba”, “Pegan”), seven major material bases (dietary essence, blood, meat, fat, bone, bone marrow, sperm), and three excretions (stool, urine, sweat), and the three major factors govern the movement of the seven major material bases and the three excretions (Zhong et al., 2022). The most essential cause of AMS is three major factors and seven major material dysfunctions (Zhang and Zhang, 1993; Feng et al., 2013).

### 5.3 Different TCM pattern types of AMS and the rules of clinical application of TCM

According to the theory of TCM and the principle of pattern differentiation and treatment, the main TCM pattern types of AMS are a pattern of Qi deficiency with blood stasis, and the main principle of treatment is activating Qi, activating blood circulation, and resolving phlegm (Feng et al., 2013; Wu et al., 2023). The clinical applications of TCM are based on compound danshen dripping pill (复方丹参滴丸), huangqi injection (黄芪注射液), shulikang capsule (舒理康胶囊), duoxuekang capsule (多血康胶囊), and xuefu zhuyu decoction (血府逐瘀汤). Commonly used botanical drugs are mainly Astragalus mongholicus Bunge (Huangqi), Salvia miltiorrhiza Bunge (Danshen), Panax ginseng C.A. Mey. (Renshen), and Rhodiola rosea L. (Hongjingtian). The TCM pattern types of AMS and the application of TCM are shown in Table 2.

**TABLE 2:** The TCM pattern types of AMS and the rules of clinical application of TCM.

TCM pattern types	Therapeutic principle	TCM preparation	Compositions	Altitude (m)	Time	Treatment vs. control	Sample size (T/C)	Main clinical outcomes	Ref
Pattern of Qi deficiency	Benefiting Qi and nourishing Yin	Hongjingtian Oral-Solution (HOS)	Rhodiola rosea L. (Hongjingtian)	>3000	7 days	HOS + Dexamethasone vs. Dexamethasone	120 (60/60)	Reduced the levels of systolic pressure, diastolic pressure and Hb, and increase the level of SaO2	(Shen, 2019, p. 6)
				3600	7 days	HOS vs. Aminophylline	200 (100/100)	Increased the cure rate	Du et al. (2004)
		Taikong Yangxin Pill (TYP)	Panax ginseng C.A. Mey. (Renshen), Citrus reticulata Blanco (Chenpi), Crataegus pinnatifida Bge. (Shanzha), Acanthopanax gracilistylus W. W. Smith (Wujiapi)	3400	7 days	TYP vs. placebo	40 (20/20)	Increased the cure rate and the level of SaO2	Jiao et al. (2023)
		Huangqi Injection (HI)	Astragalus mongholicus Bunge (Huangqi)	>3000	3 days	HI vs. Routine treatment (RT)	400 (200/200)	Significantly effective (116 cases, 58.0%), effective (84 cases, 42.0%) in HI group	Xie and Guo (2004)
Pattern of blood stasis	Activating blood circulation and removing blood stasis	Compound Danshen Dripping Pill (CDDP) (复方丹参滴丸)	Salvia miltiorrhiza Bunge (Danshen), Panax notoginseng (Burkill) F.H. Chen (Sanqi), Cinnamomum camphora (L.) Presl (Bingpian)	4000	6 days	CDDP vs. placebo group	141 (71/70)	Decreased the heart rate, myocardial oxygen consumption	Li et al. (2020b)
				>3000	>2 days	CDDP + Aminophylline vs. CDDP	120 (64/56)	Reduced the incidence of AMS (25%), compare with CDDP (43%)	Ruan (2009)
		Rongshuang capsule (RC) (溶栓胶囊)	Pheretima aspergillum (E.Perrier) (Dilong)	4300	7 days	RC vs. placebo	72 (32/40)	Increased the level of SaO2	Cui et al. (2015)
		Danhong Injection (DI) (丹红注射液)	Salvia miltiorrhiza Bunge (Danshen), Carthamus tinctorius L. (Honghua)	>3000	5 days	(DI + RT) vs. RT	76 (39/37)	Increased the overall effective rate	Fang (2011)
Pattern of Qi deficiency with blood stasis	Benefiting Qi, activating blood circulation and removing blood stasis	Shengnaokang Pill (SP) (圣脑康丸)	Salvia miltiorrhiza Bunge (Danshen), Panax notoginseng (Burkill) F.H. Chen (Sanqi), Conioselinum anthriscoides (H.Boissieu) Pimenov & Kljuykov (Chuanxiong), Hirudo (Shuizhi), Codonopsis pilosula (Franch.) Nannf. (Dangshen)	4200	7 days	SP vs. placebo	41 (26/15)	Reduced heart rate; increase the level of SaO2; decreased the incidence of AMS	Tang et al. (2013)
		Danqijing granule (DQJ) (丹芪精颗粒)	Salvia miltiorrhiza Bunge (Danshen), Astragalus mongholicus Bunge (Huangqi), Polygonatum kingianum Coll.et Hemsl. (Huangjing)	>3000	7 days	DQJ vs. HJT	80 (40/40)	Reduced serum TNF-α and IL-6 levels and improve AMS clinical symptoms	Li et al. (2023a)
		Hongjingtian Capsule; Yinxingye Tablet	Rhodiola rosea L. (Hongjingtian), Ginkgo biloba L. (Yinxingye)	3650	7 days	Hongjingtian vs. Yinxingye vs. Acetazolamide	200 (67/65/68)	Increased the level of SaO2	Gongga et al. (2015)
		Sankang Capsule (SK) (三康胶囊)	Astragalus mongholicus Bunge (Huangqi), Codonopsis pilosula (Franch.) Nannf. (Dangshen), Panax notoginseng (Burkill) F.H. Chen (Sanqi), Panax ginseng C.A. Mey. (Renshen), Cervus nippon Temminck (Lurong), Lycium barbarum L. (Gouqizi)	3200	15 days	SK vs. Danshen tablet	33 (18/15)	Increased the overall effective rate (83.3% in SC group; 46.7% in Danshen group)	Lin and Xiong (2008)
pattern of Qi stagnation and blood stasis	Activating Qi and activating blood circulation, resolving phlegm	Xingnaojing Injection (XNJ) (醒脑静注射液)	Moschus berezovskii Flerov (Shexiang), Cinnamomum camphora (L.) Presl (Bingpian), Curcuma wenyujin Y.H. Chen et C. Ling (Yujin), Gardenia jasminoides Ellis (Zhizi)	>3000	5 days	(XNJ + RT) vs. RT	78 (40/38)	Increased the overall effective rate	Fang (2008)
				>3000	7 days	(XNJ + RT) vs. RT	43 (21/22)	Increased the cure rate (66.7%) compare with the routine (22.7%)	Li and Qian (2011)
		Shulikang Capsule (SC) (舒理康胶囊)	Rosa rugosa Thunb. (Meiguihua), Juglans regia L. (Hetaoren), Lycium barbarum L. (Gouqizi), Rhodiola rosea L. (Hongjingtian), Prunus persica (L.) Batsch (Taohua), Astragalus mongholicus Bunge (Huangqi), Angelica sinensis (Oliv.) Diels (Danggui), Rheum palmatum L. (Dahuang)	3900	7 days	SC vs. placebo group	150 (50/50)	Slow the rapid heart rate, reduced the basal metabolic rate and the incidence of AMS	Niu et al., 2006b; 2006a
				>3000	15 days	SC vs. Compound Dangshen Tablet (CDT)	330 (180/150)	Total effective rate: 83.3% in SC group, 46.7% in CDT group	Lin et al. (2008)
	Benefiting Qi and protecting the heart	Shexiang Baoxin Pill (SBP)麝香保心丸	Moschus berezovskii Flerov (Shexiang), Panax ginseng C.A. Mey. (Renshen), *Bos taurus* domesticus Gmelin (Niuhuang), Cinnamomum cassia Presl (Rougui), Liquidambar orientalis Mill. (Suhexiang), *Bufo bufo* gargarizans Cantor (Chansu), Cinnamomum camphora (L.) Presl (Bingpian)	4200	4 days	SBP vs. Control	66 (26/40)	Improved headaches, panic attacks, chest tightness and shortness of breath	Yu et al. (2011)
Pattern of Qi and blood deficiency	Benefiting Qi, activating blood circulation, nourishing Yin	Compound Dangshen Capsule (CDC) (复方党参胶囊)	Codonopsis pilosula (Franch.) Nannf. (Dangshen), Glehnia littoralis Fr. Schmidtex Miq. (Beishashen), Salvia miltiorrhiza Bunge (Danshen)	5200	15 days	CDC vs. placebo	45 (30/15)	Decreased the AMS rate, improve lung ventilation function and finger motor ability	Zhang et al. (2010)
					7 days	CDC vs. placebo	45 (18/14)	Relieved headache and vomiting caused by altitude hypoxia	Zhang et al. (2005)
		Compound Dangshen Tablet (CDT) (复方党参片)	Codonopsis pilosula (Franch.) Nannf. (Dangshen), Panax quinquefolium L. (Xiyangshen), Angelica sinensis (Oliv.) Diels (Danggui), Salvia miltiorrhiza Bunge (Danshen), Glehnia littoralis Fr. Schmidtex Miq. (Beishashen)	>3000	7 days	CDT vs. RT	80 (40/40)	Total effective rate: 97.5% in CDT group, 65.0% in RT group	Li (2016)
Pattern of Qi deficiency and damp heat	Clearing heat and removing dampness, benefiting Qi for activating blood circulation	Gaoyuanan Capsule (GC)(高原安胶囊)	Panax quinquefolium L. (Xiyangshen), Astragalus mongholicus Bunge (Huangqi), Rhodiola rosea L. (Hongjingtian)	3700–4300	5 days	GC vs. placebo	50 (25/25)	Increased the level of SaO2	Cui et al. (2014)
Pattern of hyperactivity of liver-Yang	Suppressing hyperactive liver for calming endogenous wind, activating blood circulation and removing blood stasis	Xifengzhitong granule (XFZT) (息风止痛颗粒)	Uncaria macrophylla Wall. (Gouteng), Scorpio (Quanxie), Conioselinum anthriscoides (H.Boissieu) Pimenov & Kljuykov (Chuanxiong), Astragalus mongholicus Bunge (Huangqi)	4300	15 days	XFZT vs. placebo	118 (38/40)	Improved flustered, headache, and short breath	Cui et al. (2014)
N/A	N/A	Yinxingye Tablet	Ginkgo biloba L. (Yinxingye)	3696	3 days	Yinxingye vs. Acetazolamide vs. placebo	36 (12/12/12)	Reduced the rate of AMS, compare with the acetazolamide (36%)	Moraga et al. (2007)
N/A	N/A	EGb 761 Tablet	Ginko biloba L. extract (EGb 761)	5400	Twice a day	EGb 761 vs. placebo	44 (22/22)	Reduced the rate of AMS (13.6%), compare with the placebo (81.8%)	Roncin et al. (1996)
N/A	N/A	Acupuncture and moxibustion	Acupoints of Baihui, Zusanli, Qihai, Neiguan, and Zhongwan	3836	5 or 10 days	Acupuncture and moxibustion	56 (56/0)	Cured: 51 cases (91%); effective: 5 cases (9%)	Wang (1994)

Note: AMS, acute mountain sickness; CDC, compound dangshen capsule; CDDP, compound danshen dripping pill; CDT, compound dangshen tablet; DI, danhong injection; DQJ, danqijing granule; GC, gaoyuanan capsule; HI, huangqi injection; HOS, Hongjingtian oral-solution; RC, rongshuang capsule; SaO_2_, blood oxygen saturation; SBP, shexiang baoxin pill; SC, shulikang capsule; SK, sankang capsule; SP, shengnaokang pill; TYP, taikong yangxin pill; XFZT, xifengzhitong granule; XNJ, Xingnaojing Injection.

In addition, the clinical evidence of TCM for several typical AMS is summarized in Table 2. A total of 18 representative TCMs, including 6 capsules, 3 injections, 3 pills, 2 tablets, 2 granules, 1 oral solution, and 1 acupuncture were compiled and summarized. TCM formulas can improve exercise tolerance and quality of life and relieve clinical symptoms. This provides a reference and basis for clinical use and further pharmacological studies based on the results of the analysis of TCM pattern types and TCM application in AMS.

### 5.4 Frequency of commonly used Chinese botanical drugs from clinical TCM formulas

According to the data in Table 2, among a total of 33 botanical drugs used clinically, and the top 10 botanical drugs ranked by their relatively high frequency of use are shown in Table 3. Table 3 shows that the most frequently used botanical drugs were Salvia miltiorrhiza Bunge (Danshen). The type of botanical drugs is mainly invigorating qi (huangqi, dangshen, hongjingtian, renshen), activating blood and removing stasis (danshen, chuanxiong) and nourishing Yin (Beishashen, Gouqizi), as shown in Supplementary Table S1. The properties of botanical drugs are mainly warm, calm, and cold. The flavor of botanical drugs is mainly sweet, bitter, and pungent. Specific botanical drugs information is shown in Table 3.

**TABLE 3:** The information of top 10 botanical drugs with a relatively high frequency.

No.	Botanical drugs name	Latin name	Role in TCM	Flavor	Pharmacological effect	Frequency
1	Danshen	SALVIAE MILTIORRHIZAE RADIX ET RHIZOMA	Activating blood and removing stasis	Bitter	cardio-protector (Cheng, 2007)	6
2	Huangqi	ASTRAGALI RADIX	Invigorating Qi	Sweet	cardiovascular protective, immune modulatory (Chen et al., 2020; Su et al., 2021)	5
3	Dangshen	CODONOPSIS RADIX	Invigorating Qi	Sweet	anti-inflammatory, anti-tumor (Bailly, 2021; Yu et al., 2023)	4
4	Hongjingtian	RHODIOLAE CRENULATAE RADIX ET RHIZOMA	Invigorating Qi	Sweet	anti-aging, anti-hypobaric hypoxic (Zhuang et al., 2019; Hou et al., 2024)	3
5	Sanqi	NOTOGINSENG RADIX ET RHIZOMA	Hemostasis	Sweet; Bitter	inhibiting platelet aggregation (Liu et al., 2018b)	3
6	Bingpian	BORNEOLUM	Inducing resuscitation	Pungent; Bitter	neuroprotective effect and antipruritic effect (Ma et al., 2023; Luo et al., 2024)	3
7	Renshen	GINSENG RADIX ET RHIZOMA	Invigorating Qi	Sweet; Bitter	neuroprotective effect, improving markers related to blood glucose, blood pressure, and blood lipids (Chen et al., 2022; Park et al., 2022)	3
8	Beishashen	GLEHNIAE RADIX	Invigorating Qi, Nourishing Yin	Sweet; Bitter	anti-inflammatory, antioxidant, immunomodulatory (Li et al., 2023b)	2
9	Danggui	ANGELICAE SINENSIS RADIX	Blood tonification	Sweet	antifibrotic effect, anti-tumor, hematopoiesis (Qian et al., 2020)	2
10	Gouqizi	LYCII FRUCTUS	Nourishing Yin	Sweet	anti-oxidative stress and inflammatory effects (Zheng et al., 2023)	2

Note: The role in TCM, and flavors of botanical drugs are defined according to the 2020 edition of the Chinese Pharmacopoeia (Chinese Pharmacopoeia Commission Pharmacopoeia of the People’s Republic of China, 2020).

### 5.5 Mechanism of the effect of TCM for AMS

While many other TCM therapies, such as acupuncture and bloodletting, are effective in clinical applications for tissue damage caused by hypobaric hypoxia (Wang et al., 2022; Li et al., 2023a), this review focuses only on herbal treatments. Classical prescriptions or TCM formulas, as the main means of clinical treatment in modern TCM, are an important breakthrough point for the inheritance and innovative development of TCM (Xu et al., 2023). Currently, research on TCM formulas for the treatment of AMS is mainly based on the compound danshen drip pills (CDDP) (Yan et al., 2022). Several studies have confirmed that CDDP prevents hypobaric hypoxia induced tissue damage by reducing erythrocyte aggregation and hemorheology and promoting the expression of AQP1 and Nrf2 (Hu et al., 2021). Moreover, Fu et al. found that qi-long-tian formula extract attenuated the inflammatory response in AMS model rats (6000 m, exposed to a Fenglei hypobaric hypoxic chamber for 72 h) by inhibiting HIF-1a, VEGF, suppressing the expression of inflammation-associated effectors (MMP9 and TIMP1), inhibiting the expression of target genes downstream of IL-17 (Ccl2, MMP9, S100a9, and S100a8), and attenuating lung injury and immune cell infiltration (Fu et al., 2021). Our team also demonstrated that modified siwu decoction has a favorable protective effect against hypobaric hypoxia by down-regulating proteins associated with oxidative stress and the ubiquitin-proteasome system ((Superoxide dismutase 1, SOD1), (Catalase, CAT), GSTP1 and PRDX1), as well as by ameliorating hypoxia-induced upregulation of MDA and disturbances in energy and lipid metabolism (Tu et al., 2023). Meanwhile, Zeng Y et al. found that Huangqi Baihe Granules could significantly enhance lung function, reduce lung wet/dry ratio, alleviate lung tissue injury, inhibit the formation of ROS and MDA, and increase SOD activity and GSH expression, and its mechanism of action may be to inhibit the activation of TLR4/NF-κB p65/NLPR3 signaling pathway and reduce the release of downstream pro-inflammatory cytokines in lung tissue (Zeng et al., 2024).

In addition, studies on single TCMs for the treatment of AMS are mainly based on Rhodiola rosea L. (Hongjingtian), and it has been reported that chemical components salidroside can coordinate metabolic reprogramming by modulating the HIF-1α signaling pathway in AMS (Yan et al., 2021). Xie et al. found that Rhodiola rosea L. extract significantly increased SOD, GSH-Px, T-AO, and Na + -K + -ATPcase levels, decreased NF-α, IL-1β, IL-6, MDA, and LDH levels, and inhibited the NF-κB/NLRP3 signaling pathway, which further corrected the imbalance in energy metabolism and maintained the integrity of the BBB, thereby alleviates hypobaric hypoxia-induced brain injury (8000 m, exposed to a FLYDWC50-II C Fenglei hypobaric hypoxic chamber for 48 h) (Xie et al., 2022). Salidroside (Sal), the active chemical components of Rhodiola rosea L., has been reported to have potential protective effects against tissue damage caused by acute hypobaric hypoxia. Studies have shown that Sal attenuates acute hypobaric hypoxia-induced brain oxidative stress injury, inflammatory response, and BBB damage (Hou et al., 2024). Mechanically, Sal reduced the levels of ROS and MDA and the levels of TNF-α, IL-1β, and IL-6, and increased the activities of SOD and GSH-Px, thereby attenuating hypobaric hypoxia-induced pathological injury and oxidative stress (Jiang et al., 2022). In addition, Sal partially promoted energy metabolism and increased the activities of Na + -K + -ATPase and Ca2+-Mg2+-ATPase. The mechanism of action is related to inhibition of the NF-κB/NLRP3 pathway (Jiang et al., 2022).

**FIGURE 2:**
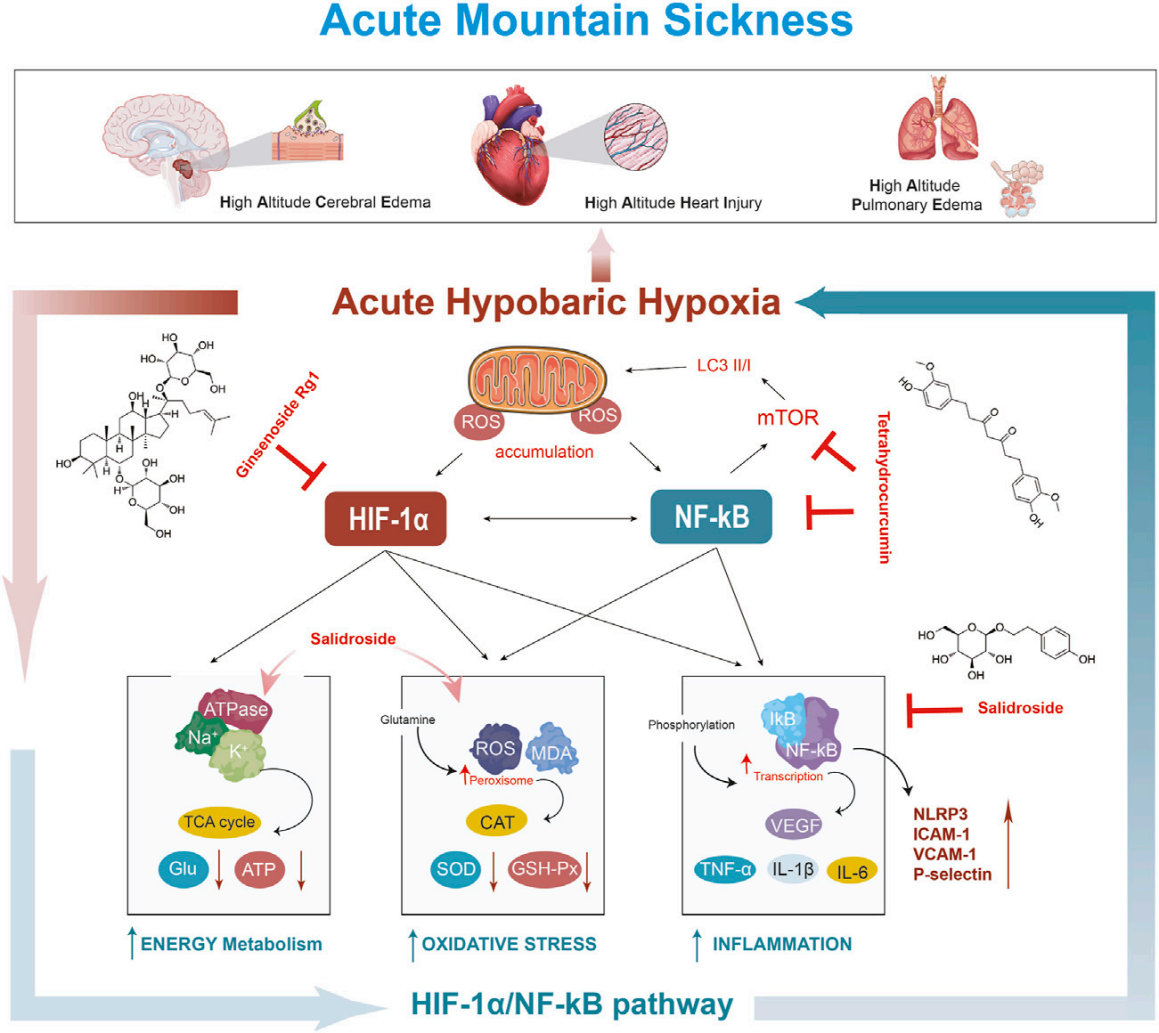
Mechanism of TCM on AMS.

Our team’s previous study found that polysaccharide extracted from Potentilla anserina L could inhibit oxidative stress and inflammatory responses to treat and prevent HACE and HAPE, and its mechanism of action may be to block the activation of the NF-κB and HIF-1α signaling pathways, which inhibits the production of downstream pro-inflammatory cytokines (IL-1β, IL-6, TNF-α, and VEGF) (Shi et al., 2019; Shi et al., 2019). Meanwhile, Pan Y et al. showed that tetrahydrocurcumin significantly reduced the expression of VEGF (AMS biomarker), MMP-9 and NF-κB, and effectively attenuated cerebral edema and inflammation induced by acute high-altitude hypobaric hypoxia (Pan et al., 2020; Nourkami-Tutdibi et al., 2023). Moreover, Zha et al. established an animal model of AMS (6000 m, 10 m/s rate of ascent, continuous exposure to hypoxia for 24 h) and found that ginsenoside Rg1 reduced the wet-to-dry ratio of lung tissues, improved the pathological changes of lung tissues, decreased the MDA content and NOS viability, elevated the CAT and Na + -K + -ATPase viability, and decreased the IL-6 and TNF-α levels (Arriaza et al., 2022). As the main active chemical component of Panax notoginseng saponins, notoginsenoside R1 attenuated inflammation and oxidative stress, reduced lung dry-to-wet ratio, alleviated the lung tissue damage, and ameliorated arterial blood gas changes in acute hypobaric hypoxia-induced HAPE rats (6000 m, exposed to a ProOx-830 hypobaric hypoxic chamber for 48 h), and its mechanism of action may be that notoginsenoside R1 promoted the activation of the ERK1/2-P90rsk-BAD signaling pathway (Pei et al., 2023). Details of TCM formulas, the specific simple botanical drugs, active components, modes of administration, experimental models, mimic altitudes, dosages, and pharmaceutical effects are shown in Table 4. The mechanism of action of TCM intervention for AMS is shown in Figure 2.

**TABLE 4:** Mechanism of the effect of TCMs for AMS.

Type	Altitude	TCM	Compositions	Experimental model	Dose/kg andday	Hypoxic time	Pharmaceutical effects	Ref
Chinese patent drug	5000 m	Compound Danshen Dripping Pill (复方丹参滴丸)	Salvia miltiorrhiza Bunge (Danshen), Panax notoginseng (Burkill) F.H. Chen (Sanqi), Cinnamomum camphora (L.) Presl (Bingpian)	SD rat	72.9 mg/kg/d (i.g.)	7 days	Promoted high altitude acclimatization of rat by improving blood oxygen saturation, reduce myocardial enzymes and restore metabolic level	Yan et al. (2022)
Chinese patent drug	6000 m	Hongjingtian Capsule	Chinese medicine preparation	SD rat	0.63, 1.26, 2.52 g/kg/d (i.g.)	7 days	Ameliorated heart injury and decreased the heart levels of TNF, VEGFA and HIF-1α in acute hypoxia-induced rats	Ma et al. (2022)
Chinese patent drug	N/A	Dazhu Hongjingtian Capsule (大株红景天胶囊)	commercial product, Chinese medicine preparation composed of R. kirilowii (Regel) Maxim	UPLC/Q-TOF-MS/MS, network pharmacology	N/A	N/A	Regulate the inflammation pathway, apoptosis pathway, and HIF-1 signaling pathway	Ou et al. (2020)
botanical drugs	8000 m 10000 m	Saussurea involucrata (Kar. et Kir.) Sch.-Bip	Extract	BALB/C mice	12.5, 25, 50 mg/kg	20 min	Decreased the mortality of animals under acute decompression conditions, improve the changes in biochemical indicators for glycometabolism and energy metabolism	Ma et al. (2011)
botanical drugs	6000 m	Rhodiola crenulata	Extract	SD rats	0.63, 1.26, 2.52 g/kg/d (i.g.)	1 day	Ameliorated heart injury and decreased the heart levels of TNF, VEGFA and HIF-1α	Ma et al. (2022)
botanical drugs	8229.6 m	Terminalia arjuna	bark extract	SD rats	150 mg/kg (p.o.)	12 h	Prevented cerebral vascular leakage, attenuated oxidative stress	Kumar et al. (2016b)

Note: HIF-1α, Hypoxia-inducible factor-1α; TNF, Tumor necrosis factor.

## 6 Scientific problems and considerations

AMS is a typical high altitude condition-specific hypoxic illness with a quick onset and progression. Even though fundamental and clinical research on AMS has advanced significantly, numerous scientific issues still need to be resolved. 1) Initial diagnosis of AMS is difficult and there is a lack of targeted treatment. Acetazolamide is currently the main drug used to treat AMS, but blind use of acetazolamide without a clear diagnosis of the disease can often lead to side effects. 2) The effects of different altitudes on the function of multiple tissues and organs of the body remain elusive. Studies have shown that different altitudes directly affect hepatic perfusion, with hepatic arterial perfusion increasing with altitude (Shi, 2011). 3) The basic experimental models of AMS are mainly animal-based, and the index parameters of model construction success are still not unified, so it is not possible to judge the success rate of the model effectively and quickly. In addition, animal experiments are time-consuming, so there is a need for cellular models for drug screening to facilitate rapid and effective drug screening.

## 7 Conclusion and perspectives

With the rapid development of tourism and mountaineering in the plateau and the construction of the plateau economy, more and more people from the plains will enter the high altitude conditions, which can cause AMS if no precautions are taken. This raises important questions about how to effectively prevent and treat AMS. Due to its multi-component, multi-target and multi-pathway action characteristics, TCM can better respond to the multi-system dysfunction caused by the complex hypobaric hypoxia environment. At the same time, TCM has long-term experience in human use, has better clinical safety, lower toxicity and side effects, and is often used in combination with chemical medicine to achieve synergistic effect. In addition, the research of TCM with both medicinal and edible values in the prevention and treatment of AMS diseases has also attracted much attention, such as Rhodiola rosea L., Tibetan Brassica rapa L., Tibetan Hippophae rhamnoides L. (HIPPOPHAE FRUCTUS) and so on. In the future, this will remain a hot spot in the research of TCM against AMS disease. According to TCM theory and practice, the etiology and pathogenesis of AMS are mainly “qi deficiency and blood stasis” and “qi stagnation and blood stasis”. TCM for treating AMS is based on “invigorating qi botanical drugs” and “botanical drugs for activating blood circulation and removing blood stasis”. At present, there is some efficacy in TCM formulas, active chemical components, and botanical drugs extracts in the treatment of AMS, and there is some research on the mechanism of action of related chemical components. Compound danshen dripping pill, Huangqi Baihe granules, salidroside, ginsenoside Rg1 and tetrahydrocurcumin have better ability in anti-AMS. These chemical components generally exert anti-AMS pharmacological effects by inhibiting the expression of VEGF (AMS marker), MDA, and pro-inflammatory factors (TNF-α, IL-1β, and IL-6), down-regulating NF-κB/NLRP3 pathway, and promoting SOD and Na + -K + -ATPase activities, which attenuates acute hypobaric hypoxia-induced tissue injury. This review focuses on the relationship between AMS and hemodynamic and hemorheological stress, changes in cytokines and inflammatory cytokines, regulation of related signaling pathways, blood-brain barrier damage, oxidative stress injury, autophagy, and summarises the establishment of AMS animal model methods. Although TCM has the potential to regulate AMS caused by hypobaric hypoxia, many studies still have some limitations. 1) The effective dose, maximum tolerated intake, and taking time for clinical treatment of TCM formulas are still uncertain. 2) In single-dose studies of TCM formula extracts or crude drugs, the pharmacological effects are still controversial. 3) As for the chemical components of TCM, except for in-depth research on components such as salidroside and ginsenoside, there are few studies on other effective chemical components of TCM. Subsequent screening and comparison of the anti-hypoxic activity of TCM chemical components is needed. 4) Due to the multi-component and multi-target characteristics of TCM, the research on TCM against acute hypobaric hypoxia in recent years has remained at the level of animal experiments, with few clinical studies. 5) There has been limited progress in translating research results into clinical applications. Future efforts should focus on exploring clinical treatment methods using TCM chemical components (salidroside, ginsenoside, etc.), such as exploring new drug delivery methods, which can improve drug targeting, stability, bioavailability, and exert its biological activity. In conclusion, this review summarizes the potential of TCM to treat AMS, but its specific mechanisms deserve further study. The solution to this problem will be beneficial to the long-term development of TCM in the treatment of body damage diseases induced by hypobaric hypoxia.

## Abbreviations

AMS, acute mountain sickness; TCM, traditional Chinese medicine; HACE, high altitude cerebral edema; HAPE, high altitude pulmonary edema; NF-κB, nuclear factor-κB; AMPK, AMP-activated protein kinase; ROS, Reactive oxygen species; SaO2, Blood oxygen saturation; Hb, Hemoglobin; RBC, Red blood cell; CDDP, Compound danshen dripping pill; TNF-α, Tumour necrosis factor-α; VEGF, Vascular endothelial-derived growth factor; MDA, malondialdehyde; SOD, Superoxide dismutase; GSH-Px, Glutathione peroxidase; mTOR, mammalian target of rapamycin; NLRP3, nod-like receptor protein 3; BBB, blood-brain barrier; LPS, lipopolysaccharide; Glu, Glucose; ATP, adenosine triphosphate; IL-6, interleukin 6.
